# No sex differences in oxygen uptake or extraction kinetics in the moderate or heavy exercise intensity domains

**DOI:** 10.1152/japplphysiol.00429.2023

**Published:** 2024-01-11

**Authors:** Maria Solleiro Pons, Lina Bernert, Emily Hume, Luke Hughes, Zander J. Williams, Mark Burnley, Paul Ansdell

**Affiliations:** ^1^Department of Sport, Exercise and Rehabilitation, Faculty of Health and Life Sciences, Northumbria University, Newcastle upon Tyne, United Kingdom; ^2^Institute of Sport and Exercise Sciences, University of Münster, Münster, Germany; ^3^Department of Respiratory Medicine, Royal Brompton Hospital, London, United Kingdom; ^4^School of Sport, Health and Exercise Sciences, Loughborough University, Loughborough, United Kingdom

**Keywords:** aerobic, female, hemoglobin, near-infrared spectroscopy, pulmonary gas exchange

## Abstract

The integrative response to exercise differs between sexes, with oxidative energy contribution purported as a potential mechanism. The present study investigated whether this difference was evident in the kinetics of oxygen uptake (V̇o_2_) and extraction (HHb + Mb) during exercise. Sixteen adults (8 males, 8 females, age: 27 ± 5 yr) completed three experimental visits. Incremental exercise testing was performed to obtain lactate threshold and V̇o_2peak_. Subsequent visits involved three 6-min cycling bouts at 80% of lactate threshold and one 30-min bout at a work rate of 30% between the lactate threshold and power at V̇o_2peak_. Pulmonary gas exchange and near-infrared spectroscopy of the vastus lateralis were used to continuously sample V̇o_2_ and HHb + Mb, respectively. The phase II V̇o_2_ kinetics were quantified using monoexponential curves during moderate and heavy exercise. Slow component amplitudes were also quantified for the heavy-intensity domain. Relative V̇o_2peak_ values were not different between sexes (*P* = 0.111). Males achieved ∼30% greater power outputs (*P* = 0.002). In the moderate- and heavy-intensity domains, the relative amplitude of the phase II transition was not different between sexes for V̇o_2_ (∼24 and ∼40% V̇o_2peak_, *P* ≥ 0.179) and HHb + Mb (∼20 and ∼32% ischemia, *P* ≥ 0.193). Similarly, there were no sex differences in the time constants for V̇o_2_ (∼28 s, *P* ≥ 0.385) or HHb + Mb (∼10 s, *P* ≥ 0.274). In the heavy-intensity domain, neither V̇o_2_ (*P* ≥ 0.686) or HHb + Mb (*P* ≥ 0.432) slow component amplitudes were different between sexes. The oxidative response to moderate- and heavy-intensity exercises did not differ between males and females, suggesting similar dynamic responses of oxidative metabolism during intensity-matched exercise.

**NEW & NOTEWORTHY** This study demonstrated no sex differences in the oxidative response to moderate- and heavy-intensity cycling exercise. The change in oxygen uptake and deoxyhemoglobin were modeled with monoexponential curve fitting, which revealed no differences in the rate of oxidative energy provision between sexes. This provides insight into previously reported sex differences in the integrative response to exercise.

## Graphical abstract

**Figure:**
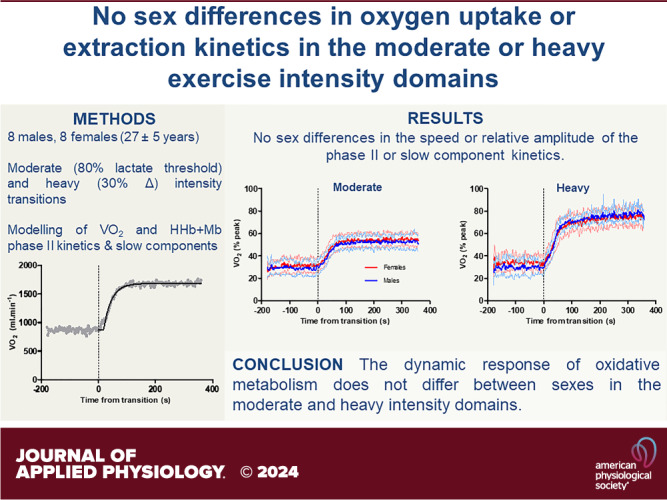

## INTRODUCTION

The transition from rest to exercise involves an integrated response from the pulmonary, cardiovascular, and muscular systems to rapidly increase the supply and utilization of oxygen for oxidative adenosine triphosphate (ATP) provision (1). The speed at which this process can occur can be quantified using pulmonary oxygen uptake (V̇o_2_) kinetics and is thought to determine metabolic stability and exercise tolerance across the spectrum of athletic performance and disease (2, 3). The V̇o_2_ response can be broken down into three phases, beginning with the initial cardiodynamic phase (phase I, 10–20 s), which represents an increased venous return via the muscle pump effect, as well as increased pulmonary blood flow (4). Thereafter, increases in pulmonary V̇o_2_ are considered to reflect increased muscle oxygen uptake in response to exercise (phase II), until the energy demand of exercise is met by oxidative phosphorylation and V̇o_2_ reaches a steady state (5). A steady-state response is attainable quickly within the moderate-intensity domain, whereas in either heavy- or severe-intensity domains, a further rise in V̇o_2_ is observed before the steady state is attained (heavy) or V̇o_2max_ is reached (severe), termed the slow component. This three-phase response is ubiquitous in exercising humans; however, the biological characteristics of the individual can influence the rates at which they occur (for review see Ref. 1).

The time constant of phase II kinetics is considered to be a crucial determinant of the decrease in contractile function experienced by the exercising individual (6, 7) and could explain previously observed sex differences in the integrative response to exercise (8). As oxidative phosphorylation does not immediately meet the demand for ATP, substrate-level phosphorylation is required (2). Intuitively, the rate at which oxidative metabolism can be upregulated at the onset of exercise is inversely linked with the accumulation of deleterious metabolites such as hydrogen ions [H^+^] and inorganic phosphate [P_i_], as well as the depletion of phosphocreatine [PCr] stores, which all interfere with excitation-contraction coupling (9). However, the relationship between the aforementioned metabolites and the upregulation of oxidative phosphorylation is multifaceted, with the progressive change in the phosphate energy state (i.e., the [ATP]/[ADP][P_i_] balance) driving the rate at which mitochondrial respiration increases, while [H^+^] accumulation concurrently inhibits anaerobic glycolysis at the onset of exercise (10, 11). Accordingly, Temesi et al. (7) demonstrated a positive correlation between the time constant (τV̇o_2_) of the phase II response and the decrease in quadriceps potentiated twitch force. Similarly, elite endurance athletes demonstrate faster τV̇o_2_ (12) and lesser declines in contractile function (13) compared with untrained individuals when exercising at similar relative intensities.

A consistent finding in studies comparing males and females exercising at the same metabolic intensity is that females experience a lesser degree of contractile impairment of the knee extensors (14–16). Previously, this has been suggested to be a result of sex differences in skeletal muscle composition, whereby females consistently demonstrate a greater proportional area of type I fibers (17, 18). The consequences of this fiber type difference are multifactorial; for instance, it is well established that type I fibers are more fatigue-resistant (19). In addition, vastus lateralis capillary density is ∼23% greater in females than in males (17) while females also demonstrate greater mitochondrial oxidative function and intrinsic respiratory rates than males of equivalent training status (20). One factor that remains unexplored is whether these physiological sex differences result in differences in the metabolic response to exercise. Conceivably, the superior aerobic phenotype of female skeletal muscle could imply that females might be able to meet the ATP demand of exercise through oxidative means faster than males. Despite this, ex vivo evidence suggests that female skeletal muscle fibers have a lower ADP sensitivity of mitochondrial respiration (21), which could result in a slower rate of oxidative phosphorylation at the onset of exercise. Therefore, in vivo assessment of V̇o_2_ kinetics would provide insight into the balance between these morphological and cellular sex differences.

The V̇o_2_ slow component is underpinned by different mechanisms to the phase II kinetics and describes the increase in V̇o_2_ during constant-load exercise. This increase in V̇o_2_ implies an impairment of efficiency and is likely an amalgamation of several concurrent physiological changes. Within skeletal muscle, the accumulation of metabolites (e.g., [Pi]) and associated contractile dysfunction is linked with the loss of efficiency (22). Of relevance here is that female skeletal muscle has consistently been demonstrated to be more fatigue-resistant (14, 15) and shows lesser increases in the surface electromyogram in states of fatigue (14, 23). Furthermore, it is well-established that females have a greater reliance on lipid metabolism during sustained exercise at similar relative work rates (24), which could be related to the more oxidative phenotype of skeletal muscle. Combined, these physiological sex differences could represent a slower loss of efficiency during constant load exercise in females, however, this remains unexplored.

Despite more aerobically suited skeletal muscle, females have lower levels of hemoglobin (25), which is thought to impair O_2_-carrying capacity during exercise (26, 27). During exercise where O_2_ delivery and utilization are both limiting factors (e.g., cycling, 28), these factors are thought to counteract each other to enable comparable relative metabolic thresholds (i.e., critical power) between the sexes (8). To date, the only investigation to systematically investigate the oxidative adjustment at the onset of exercise between sexes did so during low-intensity treadmill walking (29). Data from this study suggested faster O_2_ extraction in females, quantified as the change in deoxyhemoglobin and myoglobin (HHb + Mb) signal in near-infrared spectroscopy (NIRS), fitting with the notion that phase II V̇o_2_ kinetics are influenced by intramuscular factors in healthy humans (1). However, the demands of treadmill walking differ from those of high-intensity cycling exercise, where it has recently been argued that all levels of the O_2_ cascade are considered to be influential in determining metabolic responses to exercise (28). Thus, given the lack of evidence regarding physiological responses to exercise in females (30), it remains to be determined how sex differences in convective and diffusive contributions to O_2_ delivery mediate the rate of V̇o_2_ adjustment to exercise.

Accordingly, the present study used a multimethod approach of measuring pulmonary gas exchange and near-infrared spectroscopy simultaneously to compare the kinetics of pulmonary V̇o_2_ as well as muscle oxygen extraction in both sexes during moderate- and heavy-intensity exercises. Previously this experimental approach has been used to obtain information about O_2_ delivery and utilization to gain insights into the integrative response to exercise (31). It was hypothesized that females would demonstrate a smaller value for the phase II time constant (i.e., faster kinetics) for V̇o_2_ and HHb + Mb at the onset of exercise and a smaller slow component amplitude in the heavy-intensity domain.

## METHODS

### Ethical Approval

This study received institutional ethical approval from the Northumbria University Health and Life Sciences Research Ethics Committee (submission reference: 49189) and was conducted according to all aspects of the Declaration of Helsinki, apart from preregistration in a database. Participants volunteered for the study and provided written informed consent.

### Participants

Using the effect size for the sex difference in vastus lateralis tissue oxygenation during heavy-intensity exercise (ηp^2^ = 0.509) from the study by Ansdell et al. (15), an a priori sample size calculation determined a minimum of 14 participants (7 females and 7 males) to detect an effect (α = 0.05, power = 0.95). Therefore, eight males (means ± SD age: 27 ± 3 yr; stature: 182 ± 5 cm; body mass: 75.3 ± 10.2 kg) and eight females (means ± SD age: 27 ± 7 yr; stature: 163 ± 4 cm; body mass: 61.8 ± 5.9 kg) volunteered to take part in the study. Hormonal status was not an exclusion criterion or controlled for in this study. Female participants were tested in any phase of their menstrual cycle and there were no restrictions on hormonal contraceptive usage. This decision was based on evidence from Mattu et al. (32) who demonstrated no hormonal effects on V̇o_2_ kinetics during cycling exercise. Of the eight females, two were using combined oral contraceptive pills (Lucette and Rigevidon) and six were naturally menstruating. All participants were free from cardiovascular, respiratory, and neurological disease as well as musculoskeletal injury.

### Experimental Design

All participants visited the laboratory on three occasions across an average of 10 ± 4 days (range: 5 – 21 days). During the first visit, participants were familiarized with the experimental procedures and completed two incremental exercise tests to quantify lactate threshold and peak oxygen uptake (V̇o_2peak_). The second and third visits were identical and involved three 6-min bouts of moderate-intensity exercise (80% of lactate threshold, LT), separated by 6 min of unloaded pedaling. Thereafter, a single bout of heavy-intensity exercise (30% ΔLT – V̇o_2peak_) was performed for 30 min.

### Visit 1: Familiarization and Incremental Testing

The first visit began with participants completing a screening questionnaire to ensure inclusion criteria were met. Thereafter, participants moved onto the cycle ergometer (Velotron, SRAM, Chicago, IL), which was set up with the seat height aligned with the hip, and handlebar height set according to the participants’ comfort, these measurements were recorded and replicated for subsequent trials. The breath-by-breath gas exchange mask was then placed over the participant’s mouth and nose, and an air-tight seal was ensured before resting data were recorded. Following resting measures of pulmonary gas exchange and muscle oxygenation, participants completed five 5 min of warm-up cycling at a light intensity (60 W) at a self-selected cadence between 70–100 rpm, before commencing an incremental exercise test. The first incremental exercise test began at 75 W and increased by 25 W every 5 min. At the end of each stage, a capillary blood sample was drawn from the participants’ fingertip and immediately analyzed to determine whole blood lactate concentration (mmol.L^−1^, Biosen C-Line, EKF Diagnostics, Barleben Germany). The test was terminated once LT was identified as the first work rate at which a nonlinear increase in blood lactate concentration was observed (33), after which, participants were provided 20 min of passive rest.

Next, participants began the second incremental test with 5 min of warm-up cycling at a light intensity (60 W) at the same self-selected cadence as before. Thereafter, a ramp test beginning at 75 W commenced, with power output increasing 1 W every 2.4 s (25 W·min^−1^). This test was terminated at volitional exhaustion, defined as cadence falling >10 rpm for 5 s. Strong verbal encouragement was provided to participants throughout. A final blood lactate sample was drawn immediately after volitional exertion. The greatest 30 s average V̇o_2_ value was used to quantify V̇o_2peak_, while the final power output was used to quantify maximal ramp test power (Pmax).

### Visits 2 and 3: Square-Wave Exercise Bouts

*Visits 2* and *3* were identical and performed with a minimum of 24 h between visits. The visits involved continuous sampling of pulmonary gas exchange and near-infrared spectroscopy (NIRS) of the vastus lateralis. Trials commenced with participants performing 3 min of unloaded pedaling on the cycle ergometer. Thereafter, participants performed three repetitions of 6-min cycling bouts at 80% of the work rate associated with LT (moderate-intensity exercise), interspersed with 6 min of unloaded pedaling. Following this, participants cycled for 30 min at a work rate of 30% between the LT and V̇o_2peak_ (30%Δ, heavy-intensity exercise). Throughout this visit, participants were asked to replicate their self-selected cadence from *visit 1*, which was monitored by an experimenter throughout. Exercise intensity was altered abruptly in a “square-wave” fashion for each repetition.

Immediately following all exercises, a “physiological calibration” of NIRS signals was performed as per recommendations from Barstow (34). Participants were laid supine on a physiotherapy table, with their leg placed horizontal, and an automatic personalized tourniquet system for blood flow restriction (Delfi Medical Innovations Inc., Vancouver, BC, Canada) was placed around the thigh via a nylon cuff (11.5 cm × 86 cm, 5 mm thick), proximal to the NIRS optode. The cuff was inflated for 5 min at 120% of limb occlusion pressure to occlude blood flow, and the mean pressure was not different between males and females (120% limb occlusion pressure: 248 ± 20 vs. 249 ± 30 mmHg, *P* = 0.780). This system automatically measures limb occlusion pressure, defined as the minimum pressure required for complete restriction of arterial blood flow in a limb, and maintains the pressure during inflation to ensure consistent occlusion. Following this, pressure in the cuff was released and the hyperemic response was measured (see *Near-Infrared Spectroscopy*). This protocol allowed all NIRS data to be expressed as a % of an individual’s physiological minimum and maximum values. Physiological calibration negates any potential influence of adipose tissue thickness on NIRS data (34).

### Pulmonary Gas Exchange

During all visits, expired gas was analyzed breath-by-breath using an online system (Vyntus CPX, Jaeger, CareFusion, Germany). Oxygen (O_2_) and carbon dioxide (CO_2_) concentrations were quantified via a paramagnetic chemical fuel cell and nondispersive infrared cell, respectively. Before each test, the analyzers were calibrated using ambient air and a gas of known O_2_ (15.00%) and CO_2_ (4.97%) concentrations. Ventilatory volumes were inferred from measurement of gas flow using a digital turbine transducer (volume 0 to 10 L, resolution 3 mL, flow 0 to 15 L·s^−1^) and calibrated before each test (Hans Rudolph Inc. Kansas City).

### Near-Infrared Spectroscopy

A multidistance, continuous‐wave, single-channel NIRS (NIRO-200NX, Hamamatsu) was used to evaluate changes in vastus lateralis muscle deoxyhemoglobin and myoglobin (HHb + Mb), as well as oxyhemoglobin and myoglobin (HbO_2_ + MbO_2_) concentrations, sampled at a rate of 5 Hz. Tissue oxygenation index (TOI) was calculated as HbO_2_ + MbO_2_ ÷ [HbO_2_ + MbO_2_ + HHb + Mb] × 100. The light‐emitting probe comprised light emitting diodes operating at three wavelengths (735, 810, and 850 nm). The probe was placed on the vastus lateralis, 20 cm above the fibular head. Optodes were held in place by an elasticized bandage and covered by an opaque, dark material to avoid motion and ambient light influences.

### Data Analysis

#### V̇o_2_ kinetics.

**Figure 1.**
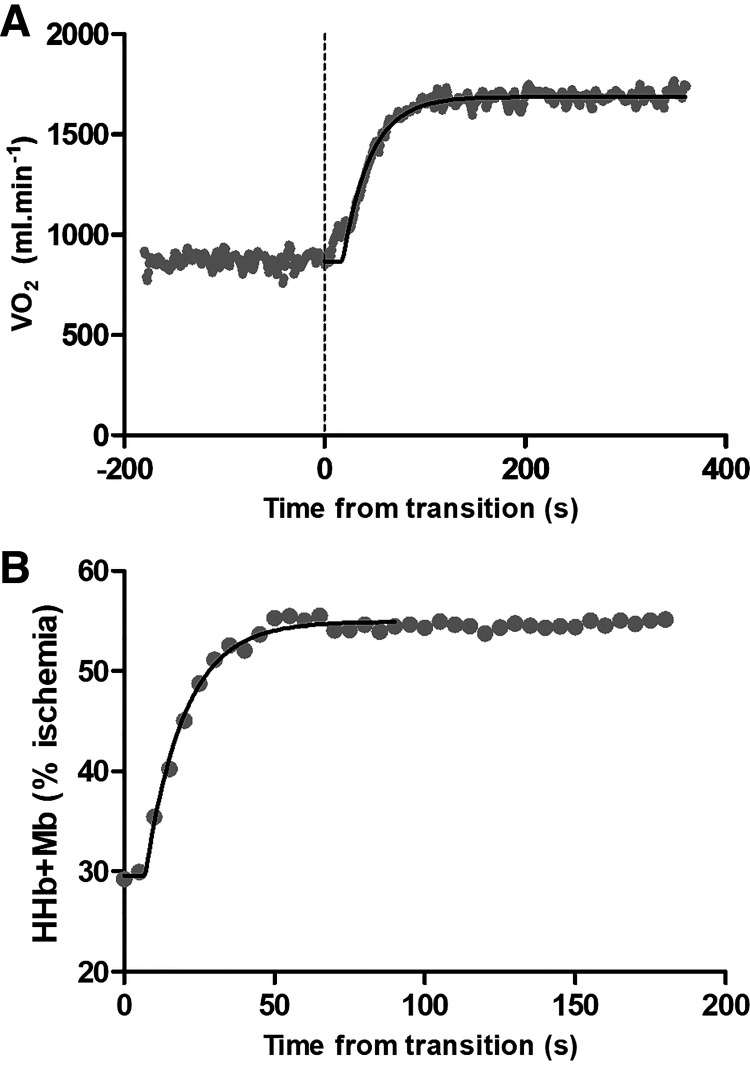
Visualization of the monoexponential curve-fitting procedures for a representative participant’s data in the moderate-intensity domain. *A* describes the V̇o_2_ data (1 Hz) and *B* describes the HHb+Mb data (0.2 Hz). HHb+Mb, deoxyhemoglobin and myoglobin; V̇o_2_, rate of oxygen consumption.

The breath-by-breath data were manually filtered to remove outlying breaths, defined as breaths deviating more than 500 mL·min^−1^ from the mean value from the preceding five breaths. Thereafter, breath-by-breath data were linearly interpolated to provide second-by-second values (see Fig. 1). The multiple repetitions of the square-wave exercise bouts were then averaged, and V̇o_2_ responses were time aligned to the onset of exercise. Data from the onset of the transition to 20 s were removed, then the resultant data were modeled with a monoexponential curve, including data from −60 to 360 s (moderate) or −60 to 120 s (heavy), with the following equation:

$${\dot{\text{V}}\text{o}}_{2}\left(t\right)={\dot{\text{V}}\text{o}}_{2}\left(\text{b}\right)+Ap\left(1-e^{-\left(\tau -\text{TDp}\right)/\tau \text{p}}\right)$$

where V̇o_2_(t) is the V̇o_2_ at time *t*; V̇o_2_(b) is the baseline V̇o_2_ measured in the 60 s preceding the transition in work rate; and *A_p_*, TD*_p_*, and τ_p_ are the amplitude, time delay, and the time constant of the phase II response, respectively. We chose to constrain the modeling of heavy-intensity domain onset kinetics to 120 s to minimize the influence of the slow component, however, this cannot be guaranteed (35). For exercise in the heavy-intensity domain, the amplitude of the V̇o_2_ slow component was determined by subtracting the phase II amplitude from the highest 30 s average of V̇o_2_ during the 30 min bout (36). To facilitate comparisons between sexes, amplitudes were also normalized to each individuals V̇o_2peak_ and end-exercise V̇o_2_ as well as being presented in L·min^−1^. The O_2_ cost of the transitions was estimated by calculating the V̇o_2_ gain (37), where the amplitude of the phase II response was divided by work rate (mL·min^−1^·W^−1^).

#### Deoxyhemoglobin kinetics.

Before curve fitting, data from NIRS were normalized to the minimum values during, and the maximum values following the 5-min arterial occlusion (38), then averaged into 1 s and 5 s bins. The multiple repetitions of the square-wave exercise bouts were then averaged, and HHb + Mb responses were time-aligned to the onset of exercise. The TD for the HHb + Mb response was determined using the 1 s averaged data as the time between exercise onset and the first point at which HHb + Mb signal started to systematically increase. This was performed for each transition individually, with all TDs averaged to provide a single value. The 5 s averaged data were then modeled with a monoexponential curve in the same manner as V̇o_2_ data, including data up to 90 s after the transition (39). Other NIRS-derived variables (TOI and HbO_2_ + MbO_2_) were quantified as 30-s averages at the following time points: the 30 s of unloaded pedaling immediately before the transition, 90 s following the transition (heavy-intensity domain only), and the final 30 s of the square wave bout of exercise.

### Statistical Analysis

Data are presented as means ± SD within the text and figures. Normal distribution of data was confirmed with the Shapiro–Wilk test. As all variables had normally distributed data, males and females were compared with independent samples *t* tests for variables with a single value or time point. For repeated-measures variables during exercise, two-way (sex × time) repeated-measures ANOVA was performed, followed by Bonferroni-corrected post hoc tests if significant main effects were observed. Effect sizes for comparisons were calculated as Cohen’s *d.* The significance level for all statistical tests was set at *P* < 0.05.

## RESULTS

### Incremental Exercise Testing

Anthropometric data and outcome variables from the two incremental exercise tests performed in the first visit are presented in Table 1. As expected, males had a greater stature and body mass than females (*P* ≤ 0.006) as well as a greater absolute V̇o_2peak_ (mean difference: 39%, *P* = 0.002). However, when V̇o_2peak_ was expressed relative to body mass, no sex difference was observed (mean difference: 14%, *P* = 0.111). Males also exercised at greater power outputs than females, with Pmax and LT being ∼30% greater in males (*P* ≤ 0.023), however when LT was expressed as a % of Pmax, no sex difference was observed (*P* = 0.373). This resulted in the power outputs for the moderate- and heavy-intensity bouts being greater in males than in females (*P* ≤ 0.023).

**Table 1.** Anthropometric data and outcome variables from incremental exercise testing

	Males (*n* = 8)	Females (*n* = 8)	*P* Value	Cohen’s d
Age, yr	27 ± 3	27 ± 7	0.734	0.117
Stature, cm	182 ± 5	163 ± 4	**<0.001**	**1.383**
Body mass, kg	75.3 ± 10.2	61.8 ± 5.9	**0.006**	**0.501**
V̇o_2peak_, L·min^−1^	3.47 ± 0.58	2.50 ± 4.42	**0.002**	**0.627**
Relative V̇o_2peak_, mL·kg^−1^·min^−1^	46.2 ± 6.6	40.5 ± 6.7	0.111	0.326
Pmax, W	328 ± 54	236 ± 43	**0.002**	**0.646**
Pmax, W·kg^−1^	4.4 ± 0.8	3.8 ± 0.5	0.118	0.253
Power at LT, W	153 ± 25	119 ± 29	**0.023**	**0.528**
LT, % Pmax	47 ± 6	50 ± 7	0.373	0.183
LT, W·kg^−1^	2.0 ± 0.2	1.9 ± 0.4	0.440	0.127
80% LT, W	123 ± 20	95 ± 23	**0.023**	**0.528**
80% LT, W·kg^−1^	1.6 ± 0.2	1.5 ± 0.3	0.440	0.110
30% Δ, W	206 ± 30	154 ± 32	**0.005**	**0.653**
30% Δ, W·kg^−1^	2.7 ± 0.4	2.5 ± 0.4	0.198	0.213

Bold type indicates statistical significance, *P* < 0.05. LT, lactate threshold; Pmax, maximal ramp test power output; V̇o_2peak_, maximal rate of oxygen consumption.

### V̇o_2_ Kinetics

**Figure 2.**
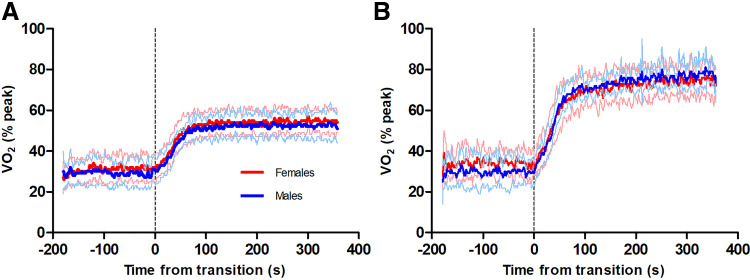
Group mean V̇o_2_ data from males (blue, *n* = 8) and females (red, *n* = 8) during moderate- (*A*) and heavy- (*B*) intensity transitions. The bold lines represent group means and the thin lines represent standard deviation. V̇o_2_, rate of oxygen consumption.

The transition from unloaded pedaling to moderate- and heavy-intensity cycling elicited an increase in V̇o_2_ (see Fig. 2), and the monoexponential curve used to describe the increase in V̇o_2_ in males and females demonstrated excellent *r*^2^ values (see Table 2).

**Table 2.** Data from the monoexponential modeling of V̇o_2_ kinetics during moderate- and heavy-intensity transitions

	Males (*n* = 8)	Females (*n* = 8)	*P* Value	Cohen’s d
*Moderate-intensity domain*				
TD, s	16.6 ± 4.1	17.9 ± 4.9	0.575	0.121
Baseline V̇o_2_, L·min^−1^	0.97 ± 0.05	0.77 ± 0.09	**< 0.001**	**1.516**
Amplitude, L·min^−1^	0.83 ± 0.19	0.60 ± 0.18	**0.024**	**0.477**
Amplitude, mL·kg^−1^·min^−1^	11.0 ± 1.6	9.7 ± 2.6	0.230	0.323
Amplitude, % V̇o_2peak_	24 ± 3	24 ± 5	0.949	0.018
V̇o_2_ gain, mL·min^−1^·W^−1^	10.2 ± 0.7	10.5 ± 1.1	0.587	0.130
τ, s	27.9 ± 7.5	24.8 ± 6.6	0.385	0.160
*r* ^2^	0.965 ± 0.032	0.947 ± 0.034		
*Heavy-intensity domain*				
TD, s	15.0 ± 4.8	14.5 ± 4.5	0.858	0.033
Baseline V̇o_2_, L·min^−1^	1.00 ± 0.06	0.85 ± 0.11	**0.005**	**0.894**
Amplitude, L·min^−1^	1.50 ± 0.38	0.96 ± 0.25	**0.005**	**0.541**
Amplitude, mL·kg^−1^·min^−1^	19.9 ± 4.7	15.6 ± 3.7	0.060	0.351
Amplitude, % V̇o_2peak_	43 ± 5	38 ± 7	0.179	0.355
V̇o_2_ gain, mL·min^−1^·W^−1^	9.4 ± 0.8	9.3 ± 0.9	0.766	0.059
τ, s	28.8 ± 7.9	27.2 ± 4.4	0.633	0.075
*r* ^2^	0.961 ± 0.031	0.927 ± 0.030		
SC amplitude, L·min^−1^	0.38 ± 0.16	0.28 ± 0.12	0.158	0.250
SC amplitude, % V̇o_2peak_	11.9 ± 6.9	10.8 ± 3.2	0.686	0.061
SC amplitude, % end exercise V̇o_2_	13.6 ± 6.5	12.9 ± 3.9	0.822	0.035

Bold type indicates statistical significance, *P* < 0.05. SC, slow component; τ, time constant; TD, time delay; V̇o_2_, rate of oxygen consumption.

In absolute units (L·min^−1^), males experienced greater amplitudes of V̇o_2_ during the phase II kinetics (*P* ≤ 0.024), however, when this was made relative to the individual (V̇o_2_ gain and % V̇o_2peak_), no sex differences were observed (*P* ≥ 0.179). Similarly, the V̇o_2_ slow component amplitude was not different between sexes in relative units (*P* = 0.686). As visualized in Fig. 2, there were no sex differences in τV̇o_2_ in either the moderate- (*P* = 0.385) or heavy- (*P* = 0.633) intensity domains. Baseline V̇o_2_ was slightly elevated at the onset of heavy-intensity exercise compared with the onset of moderate-intensity exercise for females (mean difference: 0.08 L·min^−1^, *P* = 0.028), whereas male baseline V̇o_2_ was not different (*P* = 0.312).

### Oxygen Extraction Kinetics

**Figure 3.**
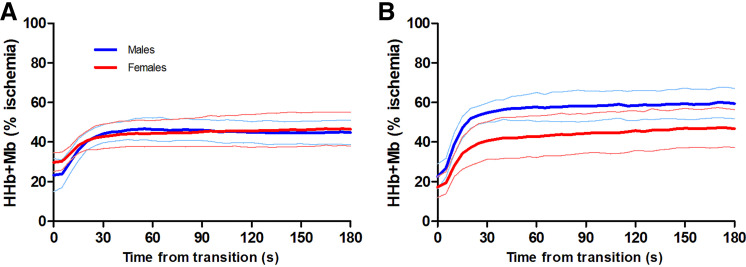
Group mean HHb+Mb data from males (blue, *n* = 8) and females (red, *n* = 7) during moderate- (*A*) and heavy- (*B*) intensity transitions. The bold lines represent group means and the thin lines represent standard deviation. HHb+Mb, deoxyhemoglobin and myoglobin.

The transition from unloaded pedaling to moderate- and heavy-intensity cycling elicited an increase in HHb + Mb concentration (Fig. 3), and the monoexponential curve used to describe the increase in HHb + Mb in males and females demonstrated excellent *r*^2^ values (Table 3). One female’s data had to be removed due to issues with the NIRS signal, resulting in *n* = 7 females being used for NIRS analyses.

**Table 3.** Data from the monoexponential modeling of deoxyhemoglobin kinetics during moderate- and heavy-intensity transitions

	Males (*n* = 8)	Females (*n* = 7)	*P* Value	Cohen’s d
*Moderate-intensity domain*				
TD, s	8.7 ± 1.5	9.6 ± 2.6	0.447	0.212
Baseline HHb+Mb, % ischemia	23 ± 8	29 ± 7	0.186	0.251
Amplitude, % ischemia	21 ± 7	17 ± 5	0.225	0.219
τ, s	8.1 ± 2.8	10.0 ± 3.8	0.274	0.260
*r* ^2^	0.974 ± 0.022	0.956 ± 0.037		
*Heavy-intensity domain*				
TD, s	6.2 ± 2.8	7.0 ± 2.3	0.582	0.101
Baseline HHb+Mb, % ischemia	23 ± 6	17 ± 7	0.146	0.332
Amplitude, % ischemia	36 ± 9	30 ± 8	0.193	0.252
τ, s	11.3 ± 3.7	12.2 ± 4.0	0.665	0.090
*r* ^2^	0.983 ± 0.011	0.979 ± 0.023		
SC amplitude, % ischemia	14 ± 5	16 ± 4	0.432	0.142

HHb + Mb, deoxyhemoglobin and myoglobin; SC, slow component; τ, time constant; TD, time delay consumption.

As shown in Fig. 3, the phase II amplitude of HHb + Mb increase in both intensity domains was not different between sexes (*P* ≥ 0.193). Similarly, there was no sex difference in the amplitude of the slow component in the heavy-intensity domain (*P* = 0.432). The time constant for phase II HHb + Mb kinetics (τHHb + Mb) was also not different between males and females in both intensity domains (*P* ≥ 0.274). Baseline HHb + Mb was slightly lower at the onset of heavy-intensity exercise for females compared with the onset of moderate exercise (mean difference: 12% ischemia, *P* = 0.002), but not for males (*P* = 0.583).

### Near-Infrared Spectroscopy

During the moderate-intensity cycling, a significant effect of sex was observed for HbO_2_ + MbO_2_ (*F*_1,13_ = 10.85, *P* = 0.006), but no time (*P* = 0.804) or sex × time interaction (*P* = 0.054) effects were observed (see Table 4). For TOI, significant time (*F*_1,13_ = 48.59, *P* < 0.001) and sex (*F*_1,13_ = 12.57, *P* = 0.004) effects were observed, but no sex × time interaction effect (*P* = 0.095). Post hoc tests revealed that females had greater values before (6%, *P* = 0.002) and during the stage (10%, *P* = 0.007). However, when TOI values were normalized as % ischemia, there was a main effect of time (*F*_1,6_ = 115.09, *P* < 0.001), but neither the main effect of sex (*P* = 0.181) or the sex × time interaction effect (*P* = 0.381) was evident.

**Table 4.** Data from near-infrared spectroscopy before and during the moderate- and heavy-intensity exercise transitions

	Moderate Intensity		Heavy Intensity		
	Unloaded Pedaling	End Stage	Unloaded Pedaling	90 s	End Stage
HbO_2_ + MbO_2_, %ischemia					
Male	57 ± 6	53 ± 7	68 ± 11*	54 ± 10*#	47 ± 10#
Female	41 ± 10	44 ± 9	51 ± 7	41 ± 4#	53 ± 8
TOI, %					
Male	69 ± 3	61 ± 5#	71 ± 4	57 ± 6#	50 ± 8#$
Female	75 ± 3*	71 ± 6*#	78 ± 4*	70 ± 8*#	69 ± 9*#
TOI, % ischemia					
Male	72 ± 4	60 ± 7#	76 ± 6	53 ± 9#	39 ± 10#$
Female	67 ± 9	53 ± 10#	77 ± 6	52 ± 9#	51 ± 17#

Males: *n* = 8; females: *n* = 7. HbO_2_ + MbO_2_, oxygenated hemoglobin and myoglobin; TOI, tissue oxygenation index. *Greater than the opposite sex (*P* < 0.05); #lower than unloaded pedaling (*P* < 0.05); $lower than 90 s.

During heavy-intensity cycling, a main effect of time was observed for HbO_2_ + MbO_2_ (*F*_2,26_ = 17.39, *P* < 0.001), as well as sex × time interaction effect (*F*_2,26_ = 15.67, *P* < 0.001), but no main effect of sex (*P* = 0.052). Post hoc tests revealed that females had lower values before (−17% ischemia, *P* = 0.004) and 90 s after the transition (−13% ischemia, *P* = 0.005), but not at the end of the stage (*P* = 0.216). Furthermore, HbO_2_ + MbO_2_ decreased in both sexes from unloaded pedaling to 90 s into the transition (*P* < 0.001), but for females, this returned to baseline by the end of the stage (*P* = 0.056), whereas males remained decreased (*P* = 0.002). For TOI, main effects of time (*F*_2,26_ = 70.21, *P* < 0.001) and a sex × time interaction effect (*F*_2,26_ = 15.67, *P* < 0.001) were observed, but no main effect of sex (*P* = 0.052). Post hoc tests revealed that females had greater values than males at all timepoints (*P* ≤ 0.005). In addition, while males demonstrated a progressive decrease in TOI at each of the three timepoints (*P* ≤ 0.001), females only decreased from unloaded pedaling to 90 s (*P* < 0.001), then no further decrease was observed at 30 min (*P* = 1.000). When TOI was normalized to % ischemia, a main effect of time (*F*_1.37,17.78_ = 49.50 *P* < 0.001) remained, however, the sex (*P* = 0.266) and sex × time interaction effect (*P* = 0.112) were not observed.

## DISCUSSION

This study aimed to compare the kinetics of V̇o_2_ and HHb + Mb during moderate- and heavy-intensity exercise in males and females. In contrast to the hypothesis, at the onset of exercise, the phase II time constants (τ) for V̇o_2_ and HHb + Mb were not different between the sexes, implying that both males and females were able to increase oxidative phosphorylation at comparable rates. In absolute units, males had larger amplitude increases than females, however, when normalized to the individuals’ maximum values, the rise in V̇o_2_ and HHb + Mb was not different. Combined, these data demonstrate that the oxidative response to exercise is not different between sexes, which provides mechanistic insight into previously observed sex differences in the integrative response to exercise.

Previous literature investigating sex differences in the onset kinetics of oxygen transport and utilization conflicts with the present data, with Beltrame et al. (29) demonstrating quicker τV̇o_2_ and τHHb + Mb in females compared with males. One potential explanation for this discrepancy could be that Beltrame et al. used a treadmill walking task, compared with cycling. In tasks where O_2_ delivery is not a limiting factor, females often outperform males. For instance, Ansdell et al. (14) showed female knee extensors had a greater relative critical torque than males during single-limb exercise. Whereas during cycling, where O_2_ delivery is a determinant of critical power (28), this metabolic threshold was not different between sexes (15). While consensus on whether O_2_ delivery does (5) or does not (40) limit τV̇o_2_ has not been reached, it is conceivable that during tasks where O_2_ delivery and utilization are both determinants in the metabolic response to exercise, the superior female skeletal muscle oxidative capacity (20) and vasodilatory response to exercise (41) are counteracted by an inferior O_2_-carrying capacity (25). Within the present data, this balance manifests as a comparable τV̇o_2_ in males and females, which agrees with data from do Nascimento Salvador et al. (42), who demonstrated no sex difference in τV̇o_2_ during a transition from unloaded pedaling to “very heavy” (60% Δ) cycling exercise.

Data from incremental exercise suggests that the poorer O_2_ delivery in females results in a greater degree of O_2_ extraction to compensate (43). The present data contradict this notion, as the amplitude of phase II HHb + Mb kinetics was not different between sexes (see Table 3). However, it is important to note that Murias et al. noted that this sex difference only occurred once incremental exercise exceeded the respiratory compensation point (i.e., the severe-intensity domain), whereas the present study compared sexes in the moderate- and heavy-intensity domains. The lack of a sex difference in the phase II amplitude for HHb + Mb kinetics contradicts previously published NIRS data that demonstrated a smaller rise in HHb + Mb and a lesser decrease in TOI in females compared with males during constant-load exercise (15). The crucial difference in methodologies used between the previous study and the present study is the application of a “physiological calibration” to negate the influence of adipose tissue thickness on NIRS signals (38). Previously, the sex difference in the rise in HHb + Mb was suggested to reflect a lower oxygen cost of muscle contraction in female knee extensors, however, the present data, with more rigorous methodologies used, refutes this. One aspect of the modeling that did demonstrate a sex difference was the reduction in HHb + Mb (and concomitant increase in V̇o_2_) baseline for the heavy-intensity transition in females, but not males. This could reflect a sex difference in how O_2_ utilization is altered by prolonged or intermittent exercise (i.e., the preceding three bouts of moderate-intensity cycling); however, the present study was not configured in a manner appropriate to answer that research question. Females did demonstrate lower HbO_2_ + MbO_2_ levels during heavy-intensity cycling, perhaps indicating a lesser O_2_ availability. This would fit with the notion that O_2_-carrying capacity is inferior in females during high-intensity exercise (26); however, the lack of difference in the speed and amplitude of HHb + Mb onset kinetics (and pulmonary V̇o_2_) implies that oxygen extraction is not negatively affected by this in the moderate- and heavy-intensity domains.

Although not measured in the present study, the sex difference in muscle fiber type, whereby females demonstrate a greater proportional area of type I fibers (17, 18), appears to have not influenced either V̇o_2_ or HHb + Mb onset kinetics in the present study. This would concur with data from Barstow et al. (44), who demonstrated no relationship between type I fiber percentage of the vastus lateralis and the time constant of phase II V̇o_2_ kinetics. In contrast, Pringle et al. (45) observed a negative correlation between type I fiber percentage and the phase II time constant in the heavy-intensity domain only. Of note is that Pringle et al. included a wide range of participants with ∼27–85% type I fibers, and when groups were split into discrete groups of low and high fiber type percentages (mean difference: 25%), the high percentage group had faster phase II kinetics. The present study was not able to quantify the sex difference in muscle fiber typology, however, previous literature has observed a 5–13% difference in type I fiber percentage of the vastus lateralis (17, 18, 46). Therefore, it could be the case that the sex difference in muscle fiber typology is not large enough to affect the phase II V̇o_2_ or HHb + Mb kinetics. Indeed, recent evidence suggests that during exercise normalized to metabolic thresholds, sex differences in muscle fiber typology do not influence fatigability (47).

Muscle fiber typology has previously been demonstrated to affect the amplitude of the slow component within the heavy-intensity domain, as individuals with a lower type I fiber percentage experience larger rises in V̇o_2_ during constant-load exercise (44, 45). It is suggested that the slow component is mechanistically underpinned by factors such as additional motor unit recruitment (48, 49) to compensate for fatigue-related changes in muscle metabolism. For instance, muscle PCr stores demonstrate a similar slow component in depletion during heavy-intensity exercise (50). Given that female knee extensors appear more fatigue-resistant and demonstrate lesser rises in the amplitude of surface electromyography during constant-load exercise (14, 15, 23), we hypothesized that the relative amplitude of the slow component would be greater in males to reflect a greater rate of metabolic disturbance. However, as is evident in Tables 2 and 3, no sex difference was observed in the relative slow component amplitude, implying that there was no difference in the metabolic response to constant-load exercise.

The lack of sex differences in either the phase II kinetics or slow component amplitude collectively suggest that the oxidative response to exercise was not different between males and females. Data on this topic are sparse, and due to the nature of methods such as magnetic resonance spectroscopy (MRS), limited to single-joint, isometric muscle contractions. Previous literature using this technique to study muscle metabolic changes during a 60-s contraction of the dorsiflexors showed no sex difference in changes in PCr, Pi, or pH (51). Data from muscle biopsies of the vastus lateralis taken before and after repeated 30-s cycling sprints suggested a greater preservation of ATP concentrations in females across a ∼60-min protocol (52); however, the authors suggested that this was likely a result of sex differences in the 20-min recovery periods, rather than metabolic differences during exercise. Accordingly, the same group observed no sex differences in the metabolic response to a single 30-s cycling sprint (53). Collectively, across multiple tasks and methodologies (MRS, biopsy, and V̇o_2_ kinetics), the data suggest that there is no sex difference in the bioenergetic response to high-intensity exercise. This information provides mechanistic insight into the sex differences in the integrative response to exercise. For instance, sex differences in fatigability have partly been attributed to a lesser accumulation of fatiguing metabolites (8, 54). It is perhaps more accurate to suggest that previously observed sex differences in fatigue during intensity-matched exercise (15, 16) are more likely due to a greater fatigue resistance of female muscle contractile apparatus, which experience similar degrees of metabolic stress as males. It is established that males and females differ in contractile properties such as calcium (Ca^2+^) kinetics of the sarcoplasmic reticulum (55), with lower Ca^2 + ^ATPase activity thought to permit a more fatigue-resistant skeletal muscle profile during equivalent exercise tasks (54). Therefore, the present study advances the contemporary understanding of sex differences in the integrative response to exercise and provides mechanistic insight into previously observed phenomena.

These data have applications across the spectrum of health and disease. For example, those prescribing steady-state exercise to improve skeletal muscle performance in athletes or patients might not need to account for the sex of their participants (56, 57). This statement should, however, be caveated by the fact that evidence regarding the influence of sex on long-term adaptation to exercise is sparse (8). Indeed, one area for further exploration is the bioenergetic response to exercise within the severe-intensity domain, where sex differences in fatigability have previously been observed (15, 16). The employment of complementary techniques to quantify O_2_ delivery (for instance, the present study did not quantify total Hb + Mb) and muscle fiber typology could also provide greater insight into the influence of sex on the O_2_ cascade in a variety of tasks.

Previous evidence from prepubertal children and adolescents suggests that boys have a lower τV̇o_2_ and V̇o_2_ slow component than girls (58), which contradicts the present findings in adults. Previous studies comparing children and adults also found that intramuscular PCr kinetics were similar in males and females regardless of age (59), implying similar oxidative capacity. Interestingly, the same study found a greater “PCr cost” (mM·W^−1^) in females compared with males suggesting a greater inefficiency in females, which contradicts the present findings. Caution is urged when comparing data from study by Willcocks et al. (59) and the present study, however, given the nature of exercise (single joint vs. whole body), the lack of matching for aerobic fitness, and the low sample size (6 males vs. 5 females).

Finally, hormonal status was not an exclusion criterion or controlled for within female participants in the present study. We based this decision on evidence from Mattu et al. (32) who demonstrated that V̇o_2_ kinetics did not differ between the follicular and luteal phases of the eumenorrheic menstrual cycle, or with oral contraceptive usage. However, we do acknowledge that the aforementioned study only investigated the moderate-intensity domain, and therefore might not apply to V̇o_2_ kinetics in the heavy-intensity domain. In the heavy-intensity domain, contributing factors to the V̇o_2_ slow component (e.g., substrate utilization) might be affected by hormonal status, although the available evidence is conflicting (60). Therefore, further research is required to determine whether endogenous and exogenous hormones influence the oxidative response to high-intensity exercise.

### Conclusions

The present study aimed to compare the oxygen extraction and uptake kinetics during moderate- and heavy-intensity cycling exercise. Contrary to our hypotheses, no sex differences were observed in either the phase II or slow component kinetics for V̇o_2_ or HHb + Mb. The lack of sex difference implies that males and females do not experience different oxidative responses to exercise, which provides mechanistic insight into previously observed phenomena such as the sex difference in fatigability. Furthermore, based on these data and others demonstrating no hormonal influences (32), we suggest that there is no rationale for the exclusion of female participants in research investigating cardiopulmonary responses to exercise.

## DATA AVAILABILITY

Data will be made available upon reasonable request.

## GRANTS

This project was supported by a Physiological Society Research Springboard Studentship awarded to M.S.P., as well as Erasmus+ funding awarded to L.B. (2020-1-DE01-KA103-005569). P.A. is supported by the UK Office for Veterans’ Affairs (G2-SCH-2022-11-12245).

## DISCLOSURES

No conflicts of interest, financial or otherwise, are declared by the authors.

## AUTHOR CONTRIBUTIONS

M.S.P., Z.W., M.B., and P.A. conceived and designed research; M.S.P., L.B., E.H., L.H., and P.A. performed experiments; L.B., E.H., L.H., Z.W., and P.A. analyzed data; M.S.P,, L.B., E.H., L.H., Z.W., M.B., and P.A. interpreted results of experiments; P.A. prepared figures; P.A. drafted manuscript; M.S.P., L.B., E.H., L.H., Z.W., M.B., and P.A. edited and revised manuscript; M.S.P., L.B., E.H., L.H., Z.W., M.B. and P.A. approved final version of manuscript.
